# Prognostic role and clinical significance of C-reactive protein-lymphocyte ratio in colorectal cancer

**DOI:** 10.1080/21655979.2021.1960768

**Published:** 2021-08-26

**Authors:** Yongsheng Meng, Chenyan Long, Xiaoliang Huang, Lihaoyun Huang, Lixian Liao, Weizhong Tang, Jungang Liu

**Affiliations:** a Division of Colorectal & Anal Surgery, Department of Gastrointestinal Surgery, Guangxi Medical University Cancer Hospital, Nanning, The People’s Republic of China; b Guangxi Clinical Research Center for Colorectal Cancer, Nanning, The People’s Republic of China; c 2^nd^ Department of General Surgery, Zhuzhou Hospital Affiliated to Xiangya School of Medicine, Hunan, The People’s Republic of China

**Keywords:** Systemic inflammatory response, C-reactive protein, lymphocyte count, overall survival, colorectal cancer

## Abstract

Systemic inflammatory response (SIRS) can be used as a potential prognostic marker in patients with colorectal cancer (CRC). The purpose of this study was to examine the predictive role of the C-reactive protein (CRP)-lymphocyte ratio (CLR) in the prognosis of CRC. We retrospectively analyzed the data of CRC patients who underwent surgery from 2004 to 2019. The clinicopathological characteristics and follow-up records were analyzed. According to a cutoff value of CLR, the patients were divided into the high and low groups. Kaplan–Meier curves and Cox proportional hazards regression model were applied to assess the overall survival (OS). Clinicopathological characteristics analysis showed that gender, age, BMI, lymphocyte count, tumor location, left- and right-sided CRC, differentiation, T stage, M stage, TNM stage, carcinoembryonic antigen (CEA), CLR, CRP, and microsatellite status were found to differ significantly between the high and low CLR groups. Kaplan–Meier curves revealed that the high CLR group had a shorter OS, and the elderly or right-sided CRC patients faced a worse prognosis. Multivariate analysis suggested that age (hazard ratio [HR]:1.011, *P* = 0.003), differentiation (HR:1.331, P = 0.000), TNM stage (HR:2.425, *P* = 0.000), CEA (HR:1.001, *P* = 0.025), CLR (HR:1.261, *P* = 0.014) were significant independent prognostic factors for OS. Subgroup analysis demonstrated that females or patients not receiving postoperative adjuvant chemotherapy with high CLR might suffer a worse prognosis. Overall, CLR can be applied as a promising prognostic marker in CRC patients and has great potential in guiding clinical work.

## Graphical abstract

**Figure:**
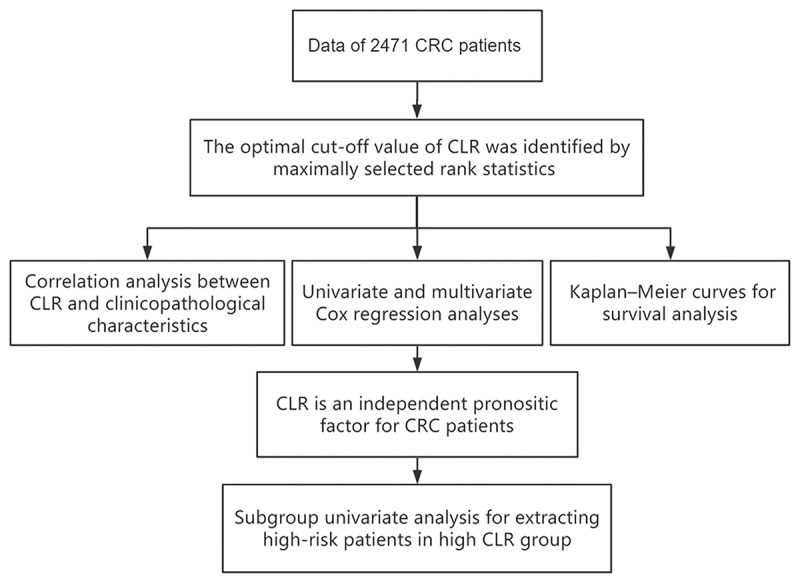

## Introduction

Colorectal cancer (CRC) is regarded as the third most common malignancy, and it is the second leading cause of cancer-related deaths worldwide [1]. Although therapeutic regimens of surgical treatment and chemotherapy have been notably improved, tumor recurrence and mortality in CRC patients remain high, and improving 5-years overall survival (OS) still a serious challenge [2,3]. The tumor–node–metastasis (TNM) stage is considered to be a major prognostic factor, and this classification system provides available information to predict prognosis [4]. However, because of the heterogeneity of this disease, the outcomes vary substantially among CRC patients, even in those with the same TNM stage. To improve the OS, it is essential to find effective biomarkers to evaluate the prognosis in CRC patients.

Systemic inflammatory response (SIRS) plays complicated and various roles in cancer. Accumulating studies have shown that SIRS can be used to predict the progression and outcomes of various malignancies [5,6]. Recent studies have demonstrated that several systemic inflammation biomarkers, such as neutrophil-lymphocyte ratio, C-reactive protein (CRP)-albumin ratio, platelet-lymphocyte ratio, were acting as predictors of prognosis in several types of malignant tumors, including CRC [5,7–9]. Therefore, based on their consequential potential in predicting prognosis, the combination of systemic inflammation biomarkers can be used for disease management and follow-up to improve the OS of tumor patients.

CRP, a phylogenetically highly conserved plasma protein, is a vital participant in the SIRS [10]. Its levels in peripheral blood rise rapidly during acute inflammation, infection, and tissue damage. Furthermore, the plasma concentration of CRP is moderately increased in cancer [11,12]. Recently, many studies have reported that increasing circulating CRP levels were related to a worse prognosis in various malignancies, such as CRC, cervical cancer, renal clear cell cancer, bladder cancer, non-small cell lung cancer, and breast cancer [13–18]. In addition, the levels of lymphocyte count are thought to be a prognostic predictor in several malignancies [19–21]. Moreover, lymphopenia is considered to strongly impact the survival of patients with metastatic solid tumors [22]. All of the above demonstrate the prognostic roles of CRP and lymphocyte count in malignant tumors. However, it is unclear how a combination of these two factors may predict prognosis in CRC.

Here, we aimed to evaluate the prognostic role and clinical significance of the CRP-lymphocyte ratio (CLR), the combination of CRP and lymphocyte count, and classify CRC patients into different risk groups for personalized treatment and management. To this end, we systematically and comprehensively analyzed the relationship between CLR and the clinicopathological characteristics of CRC patients. Furthermore, we evaluated the prognostic values for OS by focusing on our newly developed CLR as a prognostic marker in CRC patients.

## Materials and methods

### Study population

This study retrospectively analyzed data from patients who underwent surgery for CRC at the Guangxi Medical University Cancer Hospital. A total of 2471 patients with stage I-Ⅳ CRC were included from 2004 to 2019.

The inclusion criteria we used to identify eligible patients were as follows: 1) histopathological diagnosis of CRC, 2) underwent primary tumor resection, and 3) blood count and BMI data obtained preoperatively. The exclusion criteria were as follows: 1) patients with a history of familial adenomatous polyposis or hereditary non-polyposis colon cancer; 2) patients with fever during blood collection; and 3) those with other malignant tumors.

Blood test results after initial hospital admission were collected and clinicopathological characteristics including gender, age, body mass index (BMI), lymphocyte count, tumor location, left- and right-sided CRC, differentiation, T stage, N stage, M stage, TNM stage, CEA, CRP, *KRAS* phenotype, and microsatellite status were obtained from the medical records. The TNM stage was classified according to the 7th edition of the American Joint Committee on Cancer (AJCC) cancer staging manual [23]. The CLR was defined as (the C-reactive protein)/(the lymphocyte count). The primary endpoint was OS, which was calculated as the time from the surgery to the instance of the death.

All patients signed written informed consent and allowed their data to be disclosed. This study protocol was reviewed and approved by the Ethics and Human Subject Committee of Guangxi Medical University Cancer Hospital (LW2021036). All materials and methods were performed according to relevant guidelines and regulations.

### Statistical analysis

Clinicopathological characteristics, which contained continuous variables and categorical variables, were compared between the high CLR group and the low CLR group using t-test and chi-square test [24,25], respectively. The Kaplan–Meier curve was performed to compare survival outcomes [26]. Survival differences were evaluated using the log-rank test [27]. Univariate and multivariate analyses were conducted using a Cox proportional hazards regression model, and hazard ratios (HR) and 95% confidence interval (CI) were calculated [28]. All statistical tests were carried out to be two-sided and significant differences were considered at *P* < 0.05.

Statistical analyses were conducted using R statistical software (version 3.6.2.). Maximally selected rank statistics, a method for selecting the optimal splitting variable, were applied to obtain the optimal cutoff value of CLR by using the ‘survminer’ software package [29]. Univariate and multivariate analyses were performed using the ‘survival’ software package (Version: 3.1–8).

### Results

In this study, we hypothesized that CLR has great potential in predicting the prognosis of CRC patients. For this purpose, we compared the correlation between CLR and clinicopathological characteristics. Survival analysis identified that CLR could significantly distinguish the prognosis of CRC patients. Then, univariate analysis and multivariate analysis proved that CLR is an independent prognostic factor for CRC patients. Subsequently, subgroup univariate analysis further screened out high-risk groups through risk stratification. Overall, we found that CLR is a strong indicator for predicting the prognosis of CRC patients.

### Relationship between CLR and clinicopathological characteristics of CRC patients

**Figure 1.**
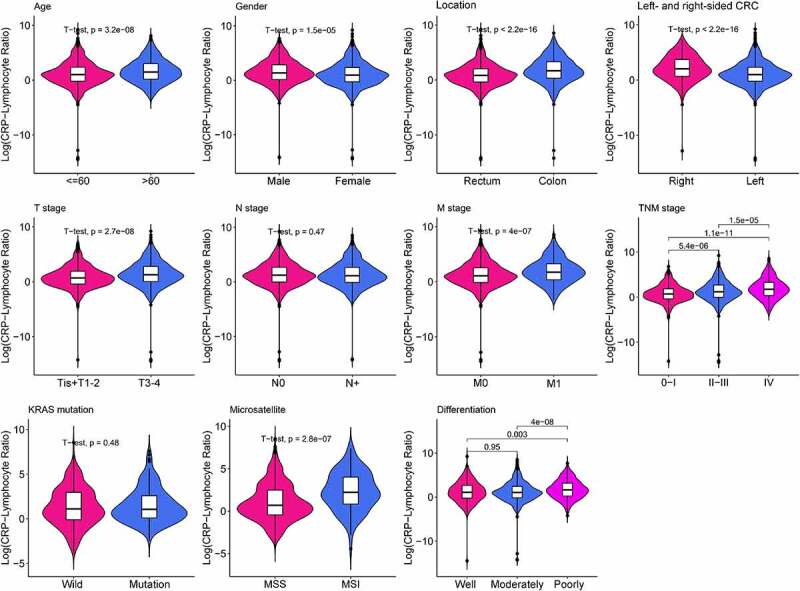
Violin plots show the relationship between CLR and clinical features. The clinical features include age, gender, tumor location, left- and right-sided CRC, T stage, N stage, M stage, TNM stage, *KRAS* mutation, microsatellite, and differentiation. Except for N stage and *KRAS* mutation, other parameters are statistically significant

Medical record for a total of 2471 CRC patients were retrospectively collected. Based on the correlation between CLR and OS in CRC patients, the optimal cutoff value for CLR was calculated as 5. The patients were divided into two groups: low CLR group with CLR≤5 (n = 1742) and high CLR group with CLR> 5 (n = 729), which could optimally predict the prognosis in CRC patients. The clinicopathological characteristics of these patients are listed in Figure 1 and Table 1. Gender, age, BMI, lymphocyte, location, left- and right-sided CRC, differentiation, T stage, M stage, TNM stage, CEA, CLR, CRP, and microsatellite status were found to be significantly different (*P* < 0.05) between the high and the low CLR groups, except for N stage and *KRAS* status (*P* > 0.05).

**Table 1.** The relationship between CLR and clinicopathological features of CRC patients

Characteristics	Case	CLR		*P
		Low(CLR≤5)	High(CLR>5)	
Total	2471	1742	729	
Gender (%)				0.001*
Male	971(39.3)	722(41.4)	249(34.2)	
Female	1500(60.7)	1020(58.6)	480(65.8)	
Age (year, mean (SD))	2471	57.26(12.74)	59.56(13.14)	<0.001*
BMI (kg/m^2^,mean (SD))	2471	22.14(3.08)	21.81(3.24)	0.015*
Lymphocyte (1 × 10^9^,mean (SD))	2471	1.87(0.63)	1.51(0.56)	<0.001*
Location (%)				<0.001*
Rectum	1221(49.4)	756(43.4)	465(63.8)	
Colon	1250(50.6)	986(56.6)	264(36.2)	
Left- and Right-Sided CRC (%)				<0.001*
Left	1888(76.4)	1417(81.3)	471(64.6)	
Right	583(23.6)	325(18.7)	258(35.4)	
Differentiation (%)				<0.001*
Well	222(8.9)	158(9.1)	64(8.8)	
Moderately	1576(63.8)	1154(66.2)	422(57.9)	
Poorly	673(27.2)	430(24.7)	243(33.3)	
T stage (%)				<0.001*
Tis+T1-2	401(16.2)	326(18.7)	75(10.3)	
T3-4	2070(83.8)	1416(81.3)	654(89.7)	
N stage (%)				0.882
N0	1362(55.1)	958(55.0)	404(55.4)	
N1-2	1109(44.9)	784(45.0)	325(44.6)	
M stage (%)				<0.001*
M0	2095(84.8)	1516(87.0)	579(79.4)	
M1-2	376(15.2)	226(13.0)	150(20.6)	
TNM stage (%)				<0.001*
0	23(0.9)	17(1.0)	6(0.8)	
I	287(11.6)	238(13.7)	49(6.7)	
II	938(38.0)	637(36.6)	301(41.3)	
III	847(34.3)	624(35.8)	223(30.6)	
IV	376(15.2)	226(13.0)	150(20.6)	
CEA (ng/ml,mean (SD))	2471	16.02(60.98)	35.65(117.33)	<0.001*
CLR (mean (SD))	2471	1.72(1.26)	25.62(39.62)	<0.001*
CRP (mean (SD))	2471	3.04(2.37)	33.68(38.14)	<0.001*
KRAS (%)				0.311
Wild	323(13.1)	220(12.6)	103(14.1)	
Mutation	171(6.9)	124(7.1)	47(6.4)	
NA	1977(80.0)	1398(80.3)	579(79.4)	
Microsatellite status (%)				<0.001*
MSI	101(4.1)	54(3.1)	47(6.4)	
MSS	1000(40.5)	741(42.5)	259(35.5)	
NA	1370(55.4)	947(54.4)	423(58.0)	

The chi-square test was used for categorical variables and the t-test was used for continuous variables.

*P value ≤ 0.05 was considered significant.

BMI: body mass index; CEA: carcinoembryonic antigen; CLR: C-reactive protein-lymphocyte ratio; CRP: C-reactive protein; KRAS: mutation in Kirsten rat sarcoma viral oncogene homolog; MSI: microsatellite instability; MSS: microsatellite stability.

### Kaplan–Meier curves for OS in CRC patients stratified by CLR

**Figure 2.**
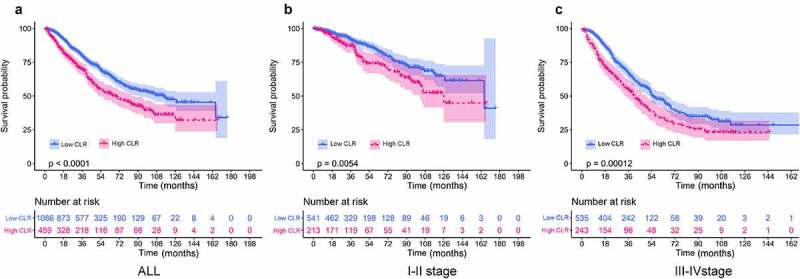
Kaplan–Meier curves of overall survival in patients stratified by CLR ratio. (a) Kaplan–Meier curves of overall survival in all CRC patients. The value of CLR above 5 means high level group and vice versa. (b) Kaplan–Meier curves of overall survival in stage I–II CRC patients. (c) Kaplan–Meier curves of overall survival in stage III–IV CRC patients

To analyze the role of CLR as a prognostic predictor in CRC patients, we used Kaplan–Meier curves to evaluate the OS in patients stratified by CLR. The Kaplan–Meier curves for OS at different TNM stage patients are shown in Figure 2. OS in the high CLR group was shorter than that in the low CLR group in all CRC patients irrespective of the TNM stage (*P* < 0.0001) (Figure 2(a)). Moreover, shorter OS was found in the high CLR group in the stage I–II CRC patients (*P* < 0.0054) (Figure 2(b)), as well as in the stage III–IV CRC patients (*P* < 0.00012) (Figure 2(c)). The above analysis showed that patients in the high and low CLR groups had significantly different outcomes. Obviously, the difference in CLR level can accurately distinguish the prognosis of patients. Moreover, our results indicated that CLR could be a strong predictor for the prognosis in CRC patients.

### Impact of CLR combined with clinical characteristics on patient prognosis

**Figure 3.**
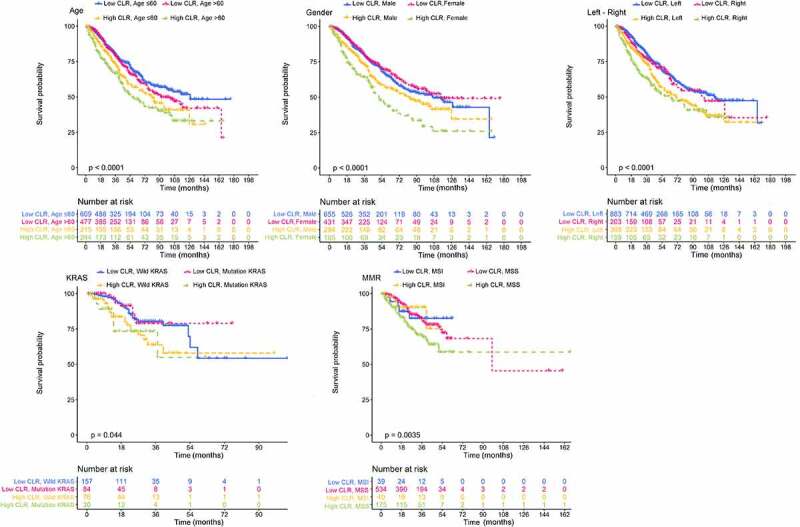
Impact of CLR combined with other clinical features on prognosis. Age subgroup: high CLR, age>60 group means poor prognosis. Gender subgroup: high CLR, female group means poor prognosis. Left-right subgroup: high CLR, right group means poor prognosis. *KRAS* subgroup: high CLR, mutation *KRAS* group means poor prognosis. MMR subgroup: high CLR, MSS group means poor prognosis

We used Kaplan–Meier curves to assess the prognosis among different clinical subgroups of CRC patients by dividing patients into subgroups based on the combination of CLR with different clinical characteristics (Figure 3). In the age subgroup defined by the combination of CLR with either age≤60 or age>60, Kaplan–Meier curves showed a significantly worse prognosis in the high CLR and age>60 subgroup (*P* < 0.001). In the gender subgroup defined by the combination of CLR with either male or female, Kaplan–Meier curves showed a significantly worse prognosis in the high CLR and female subgroup (*P* < 0.001). In the left-right subgroup defined by the combination of CLR with either left-sided CRC or right-sided CRC, Kaplan–Meier curves showed a significantly worse prognosis in the high CLR and right subgroup (*P* < 0.001). In the *KRAS* subgroup defined by the combination of CLR with either wild-type *KRAS* or mutation *KRAS*, Kaplan–Meier curves showed a significantly worse prognosis in the high CLR and mutation *KRAS* subgroup (*P* < 0.044). In the mismatch repair (MMR) subgroup defined by the combination of CLR with either microsatellite instability (MSI) or microsatellite stability (MSS), Kaplan–Meier curves showed a significantly worse prognosis in the high CLR and MSS subgroup (*P* < 0.0035). Based on these results, the combination of CLR with clinical characteristics showed a great impact on the prognosis when CRC patients were divided into different clinical subgroups.

### Univariate and multivariate analyses of the potential predictive factors for OS

**Figure 4.**
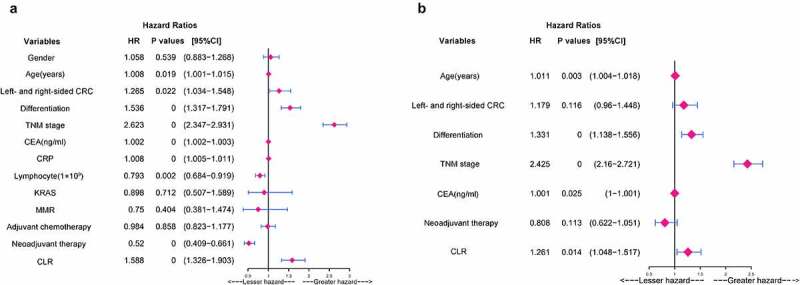
Univariate and multivariate analyses of the potential predictive factors for overall survival. (a) Univariate analysis of clinicopathological factors for overall survival. (b) Multivariate analysis of clinicopathological factors for overall survival

In univariate analysis, age (*P* = 0.019), left- and right-sided CRC (*P* = 0.022), differentiation (*P* = 0.000), TNM stage (*P* = 0.000), CEA (*P* = 0.000), CRP (*P* = 0.000), lymphocyte count (*P* = 0.002), neoadjuvant therapy (*P* = 0.000), CLR (*P* = 0.000) were significant potential predictive factors for the prognosis in CRC patients (Figure 4(a)). In multivariate analysis, age (HR: 1.011, 95%CI: 1.004–1.018, *P* = 0.003), differentiation (HR: 1.331, 95%CI: 1.138–1.556, *P* = 0.000), TNM stage (HR: 2.425, 95%CI: 2.16–2.721, *P* = 0.000), CEA (HR: 1.001, 95%CI: 1–1.001, *P* = 0.025), and CLR (HR:1.261, 95%CI:1.048–1.517, P = 0.014) were significant independent prognostic factors for OS (Figure 4(b)).

### Subgroup analysis of CRC patients stratified by baseline features in the high CLR group

**Figure 5.**
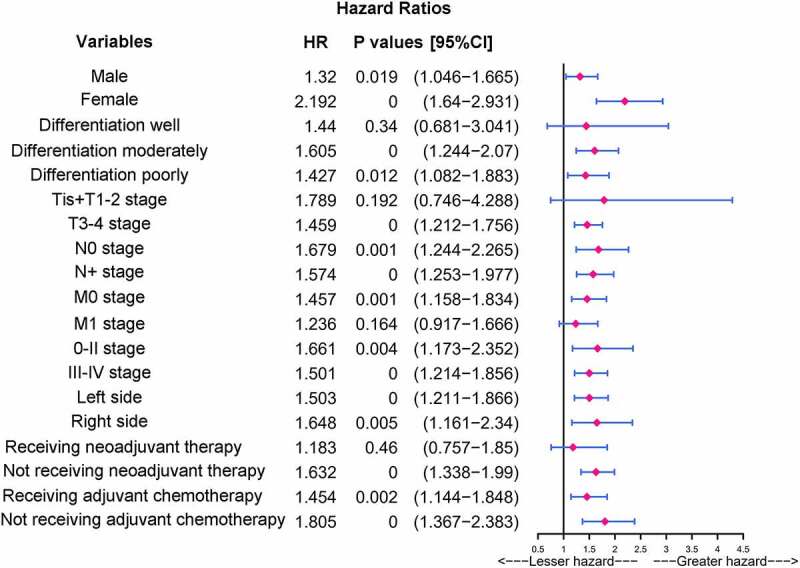
Subgroup analysis of CRC patients stratified by baseline features in the high CLR group. Univariate analysis of the CLR values of baseline features for overall survival in the high CLR group

As indicated by the results reported above, CLR was a significant independent prognostic factor for OS and the high CLR group had a poorer prognosis. To further stratify the risk of different CRC patients and identify the factors affecting the patient prognosis, we focused on the relationship between CLR and outcomes in each subgroup depending on baseline features of the patients using univariate analysis in the high CLR group. The CLR levels were significantly associated with prognosis in patients with different baseline features, including male, female, differentiation moderately, differentiation poorly, T3-T4 stage, N0 stage, N+ stage, M0 stage, 0-II stage, III–IV stage, left side, right side, not receiving neoadjuvant therapy, receiving adjuvant chemotherapy, and not receiving adjuvant chemotherapy in the high CLR group. In particular, female (HR: 2.192, CI: 1.64–2.931, *P* = 0.000), or patients not receiving adjuvant chemotherapy (HR: 1.805, CI: 1.367–2.383, *P* = 0.000) with high CLR were found to have a worse prognosis (Figure 5).

## Discussions

It is increasingly accepted that chronic inflammatory response plays a key role in the occurrence, development, and progression of tumors [30]. Chronic inflammation will accelerate the development of cancer, and the inflammation caused by tumors will produce a ‘snowball effect,’ allowing cancer to continue to progress [31]. Under these considerations, we believe that a combination of inflammation-related indicators have a significant potential in predicting the initiation and development of tumors and patient prognosis. Thus, we constructed a systemic inflammatory indicator, CLR, the combination of CRP and lymphocyte count, to predict the prognosis in CRC patients.

In the present study, we conducted an in-depth and comprehensive study on the relationship between CLR and the clinicopathological characteristics and prognosis in CRC patients. First, significant associations between the CLR and clinicopathological characteristics were observed. High CLR group was found to be associated with the older age, female, right-sided CRC, lower differentiation, advanced T stage, advanced M stage, and advanced TNM stage. Then, survival analysis demonstrated that CLR could effectively classify different TNM stage patients into different prognostic groups, and the high CLR group had a poor prognosis in the all stages, I–II stage, and III–IV stage, respectively. When CLR was combined with clinical characteristics on prognosis in subgroup analysis, different clinical subgroups including age, gender, location, *KRAS*, and MMR subgroups were able to effectively differentiate prognostic outcomes among CRC patients. In addition, univariate and multivariate analyses revealed that CLR, like the common tumor marker TNM stage and CEA, was an independent prognostic factor for OS. Furthermore, subgroup analyses based on high CLR group demonstrated that female or patients not receiving adjuvant chemotherapy CRC had a worse prognosis.

Since CRC is a heterogeneous disease, CRC patients with different TNM stages have different clinical outcomes, even the patients with the same TNM stage, which manifests that using the TNM stage alone predicting CRC patient survival may not be accurate enough. It is worth noting that compared with the low CLR group, CRC patients in the high CLR group had a shorter OS in the all stages, I–II stage, and III–IV stage, respectively. In addition, CLR was considered to be an independent prognostic factor for OS. Thus, by dividing the patients into different risk groups, the CLR can accurately predict the prognosis in patients with different TNM stage, which can be used as a supplement to the TNM stage. Interestingly, our study showed that the levels of CLR were associated with left- and right-sided CRC, and CLR can divide the left- and right-sided CRC into different prognostic subgroups, high CLR and right-sided CRC patients might face a shorter OS. Indeed, there are significant differences between the left- and right-sided colon. The left-side colon is thought to be originated from the embryonic hindgut, while the right-side colon originates from the embryonic midgut [32]. Thus, the result of the differences in biology may be caused by the different origins between the left- and right-sided colon. Therefore, left- and right-sided CRC might substantially differ in prognosis [32,33]. Furthermore, this study might demonstrate the different genetic and molecular mechanisms of the left- and right-sided CRC at the CLR level. In addition, we found that the elderly, who were over 60 years old, had a worse prognosis, especially in the high CLR group. Considering that the elderly patients face poor outcomes, they can be stratified into different risk groups based on the levels of CLR, and more attention needs to be paid to the treatment and follow-up for this group of patients.

Current studies have shown that the combination of inflammation indicators has effectively demonstrated the prognostic value in patients with malignant tumors. The CLR constructed in this study can accurately predict the prognosis in CRC and is proved to be an independent prognostic factor in multivariate analysis. Moreover, the combination of different clinical characteristics of patients with CLR can stratify the patients into different risk groups, thus more accurately predicting the prognosis in CRC patients with different clinical characteristics. Furthermore, since peripheral CRP and lymphocyte count can be obtained easily, such test is noninvasive, rapid, and inexpensive. Therefore, CLR has the potential to be widely used in clinical practice.

Although our research on CLR is profound, there are still several limitations in our study. First of all, this was a single-center study, which makes the data may not be fully convincing, even if our study is rigorous. Secondly, our study was a retrospective study, inevitably causing certain selectivity bias. Furthermore, although we collected sufficient data from CRC patients, the data become limited when divided into high and low CLR groups, limiting the general application of CLR. Therefore, a multi-center, prospective, large-scale study will provide higher universality and applicability of CLR.

## Conclusion

In summary, to our knowledge, few studies have reported the prognostic value of CLR in CRC patients. In this study, CLR was used to divide CRC patients into the high and low CLR groups. The high CLR group had a poorer prognosis, and CLR was an independent prognostic factor. Therefore, CLR can be used as a potential prognostic marker to predict CRC patient prognosis.

## Data Availability

All original data included in the manuscript are available upon reasonable request.

## Highlights

1) CLR is a significant independent prognostic factor for OS.

2) Females or patients not receiving postoperative adjuvant chemotherapy with high CLR suffer a worse prognosis.

3) CLR can be applied as a promising prognostic marker for CRC patients and has great potential in guiding clinical work.

## Abbreviations

SIRS, systemic inflammatory response; CRC, colorectal cancer; CLR, C-reactive protein-lymphocyte ratio; OS, overall survival; CEA, carcinoembryonic antigen; CRP, C-reactive protein; TNM, tumor–node–metastasis; BMI, body mass index; AJCC, the American Joint Committee on Cancer; HR, hazard ratios; CI, confidence interval; KRAS, mutation in Kirsten rat sarcoma viral oncogene homolog; MMR, mismatch repair; MSI, microsatellite instability; MSS, microsatellite stability.
